# Persistent Peril: Recurrent Deep Vein Thrombosis and Pulmonary Embolism in a Patient With Protein S Deficiency Despite Optimal Anticoagulation Therapies

**DOI:** 10.7759/cureus.60517

**Published:** 2024-05-17

**Authors:** Ali Ghorbani, Jamie Greathouse, Sina Bakhshaei, Aida Ghorbani, Kurosh Zamiri, Lauren Ho, Andrew Ho

**Affiliations:** 1 Internal Medicine, Southwest Healthcare, Temecula, USA; 2 Internal Medicine, Temecula Valley Hospital, Temecula, USA; 3 Internal Medicine, Southern California Medical Education Consortium, Temecula Valley Hospital, Temecula, USA; 4 Neurology, UCLA School of Medicine, Los Angeles, USA; 5 Biology, University of California Los Angeles, Los Angeles, USA; 6 Cardiology, Temecula Valley Hospital, Temecula, USA

**Keywords:** apixaban, recurrent dvt, hereditary protein s deficiency, hereditary thrombophilia, hypercoagulable state, acute hypoxemic respiratory failure, recurrent pe, deep vein thrombosis (dvt), right heart strain, submassive pulmonary embolism

## Abstract

The clotting system has evolved as an adaptive mechanism to prevent blood loss during vascular damage. However, the intricate nature of the clotting cascade and the complexities of human life can sometimes lead to the unnatural activation of this delicate cascade. This can result in blood clot formation within the cardiovascular system, contributing to a wide range of pathological conditions. Abnormal intravascular coagulation most commonly occurs in the deep veins of the lower extremities, and can emboli to other organs, hence, it is termed "venous thromboembolism" (VTE).

In this report, we introduce a challenging case of VTE that poses a dilemma for current medical management. The patient with possible protein S deficiency underwent various guideline-directed medical treatments, yet experienced recurrent VTE episodes, including deep vein thrombosis (DVT) and pulmonary embolism (PE), leading to hospital readmissions. This case report sheds light on our challenges in effectively treating VTE.

## Introduction

Among the various types of pathologic intravascular coagulation, deep vein thrombosis (DVT) and pulmonary embolism (PE) garner significant clinical attention. The term "venous thromboembolism" (VTE) specifically refers to the formation of a clot within the deep veins, commonly occurring in the lower extremities, with PE representing the most feared complication stemming from DVT. Moreover, the location of DVT markedly influences the risk of embolization. A DVT that occurs more proximally carries a heightened risk. Nevertheless, even isolated calf DVT, associated with a very low immediate risk of PE, poses a higher risk of extending into proximal veins and subsequently increasing the likelihood of PE. The risk increases with the extent of the DVT, particularly if it measures over 5 cm in length, and also with the vein involved, especially if the thrombus width is 6 mm or more. Annually, approximately 2 million new cases of DVT are reported in the US, with approximately 30% of proximal DVTs (such as popliteal (POP), femoral, and pelvic veins) progressing to PE. Consequently, between 60,000 to 300,000 individuals in the US succumb to PE each year [1]. Despite advances in managing VTE using direct oral anticoagulants (DOACs), vitamin-K antagonists (VKAs), and low molecular weight heparin as guideline-directed medical therapies (GDMT), the risk of recurrent VTE during anticoagulant therapy is uncommon enough to warrant investigating the cause of the recurrence.

 In a meta-analysis, involving 26,872 VTE patients in modern acute VTE treatment trials (with most treated for six months and one trial allowing treatment for up to one year), Van Es and colleagues demonstrated that the risk of recurrent VTE during the acute treatment phase was approximately 2% in patients treated with DOACs and VKAs [2]. In a long-term secondary prevention network meta-analysis, recurrent VTE occurred at a rate of 1.2 per 100 person-years with standard-intensity VKA, and similar rates were observed with other anticoagulant options [2]. Therefore, most patients undergoing acute, sub-acute, and long-term VTE treatment phases do not experience recurrent VTE, prompting us to question why some patients develop this complication. Furthermore, data on the risk of failure to multiple anticoagulants, as seen in this patient, has not been extensively studied. This gap applies to patients with both acquired and hereditary thrombophilia.

## Case presentation

**Figure 1:**
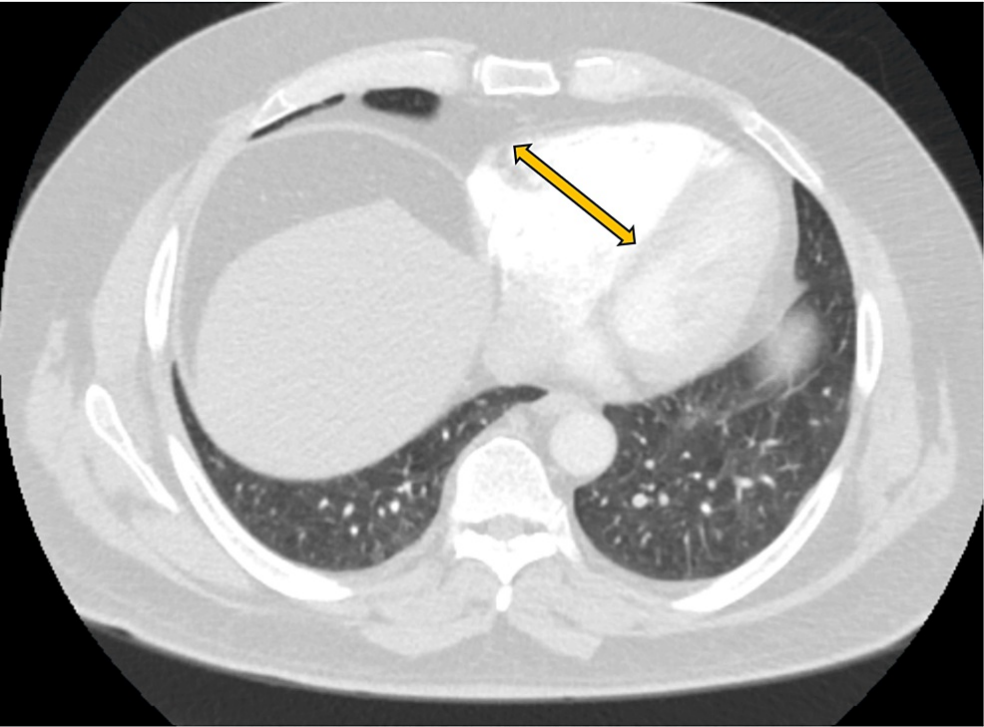
CT angiogram of the chest at the right ventricular level shows an enlarged RV (arrow) (2021) RV: Right ventricle

**Figure 2:**
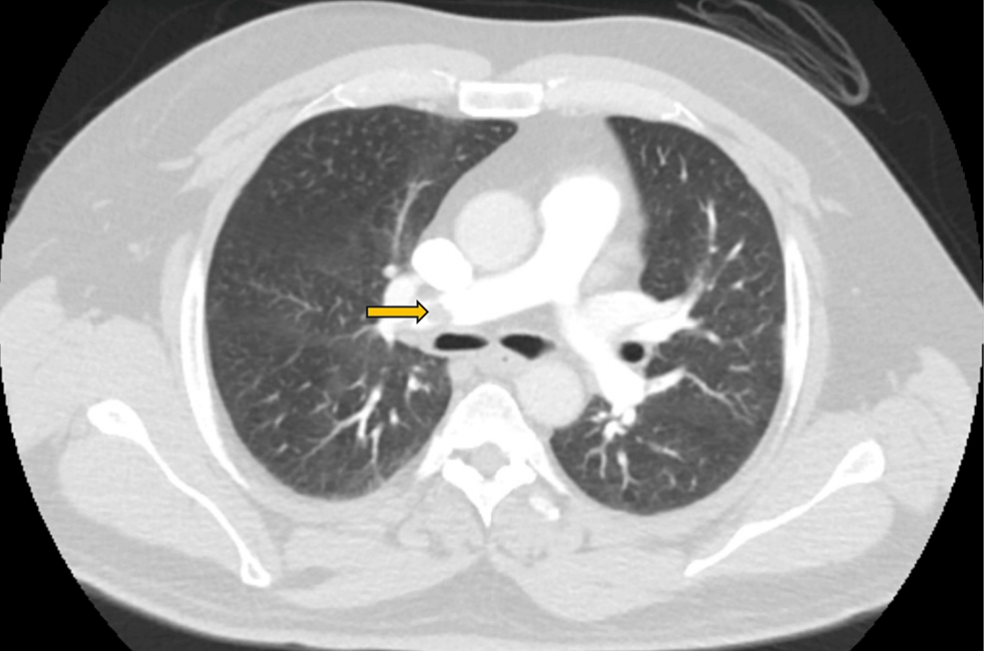
CT angiogram of the chest at the level of main pulmonary artery branches shows emboli (arrow) (2021)

The patient, a 67-year-old male, presented to the emergency department (ED) with progressively worsening shortness of breath over several days. His medical history revealed a previous diagnosis of chronic DVT. Family history was negative for any blood or coagulation disorders. The initial DVT episode had occurred about nine years ago, for which he had received treatment with rivaroxaban for six months. Subsequently, upon completing the treatment course, he had experienced a recurrence of DVT in following year, prompting the initiation of indefinite rivaroxaban therapy. Three years prior to this admission, the patient had presented to the ED again and was diagnosed with a new-onset PE (refer to Figures 1, 2).

**Figure 3:**
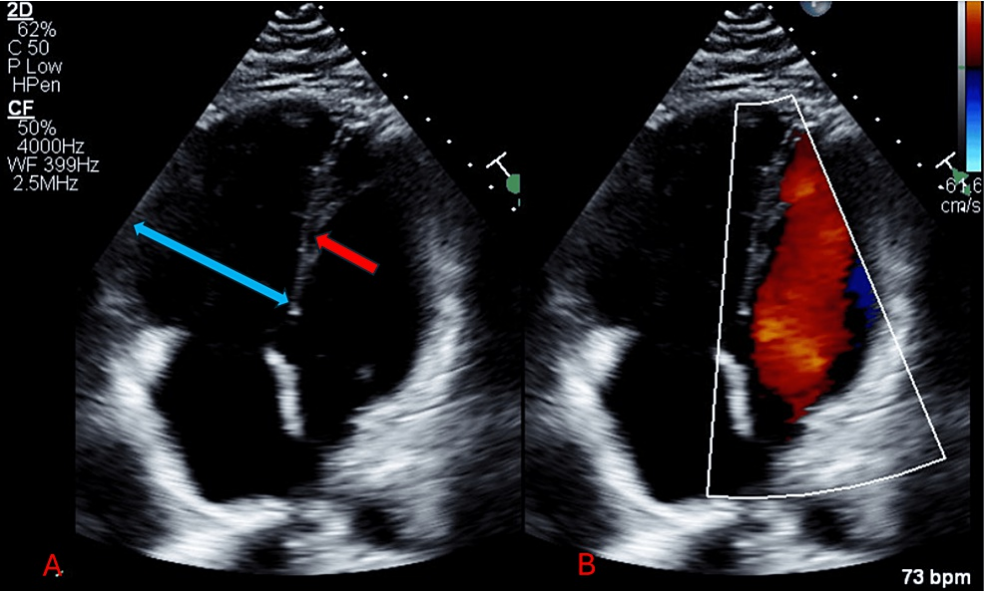
A) Echo showing a dilated RV (blue arrow) indicative of right heart strain and a flattened interventricular septum (red arrow); B) The left ventricle displays a normal configuration with a flattened interventricular septum (from last admission in 2024) Echo: Echocardiogram; RV: Right ventricle

The diagnosis was confirmed based on the findings from CT angiography (CTA) of the chest, revealing extensive bilateral pulmonary artery filling defects originating from the right main and left lobar pulmonary arteries, which extended distally (Figure 2). An echocardiogram (Echo) conducted at the time indicated right ventricle (RV) dilatation and tricuspid regurgitation, with a measured right ventricular systolic pressure (RVSP) of 30 mmHg (Figure 3).

**Figure 4:**
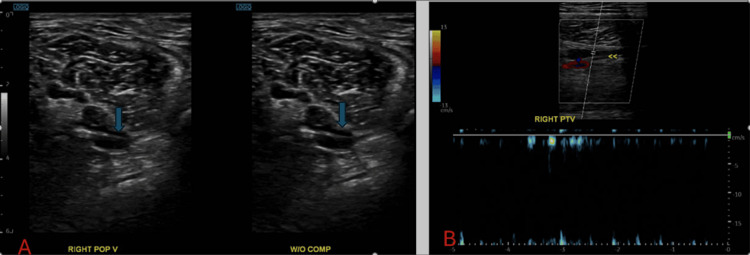
A) Doppler US of lower extremities veins shows non-compressible POP vein (arrows); B) US of right PV. The vein here is non-compressible (from last admission in 2024) US: Ultrasound; POP: Popliteal; PTV: Posterior tibial vein

A bilateral lower extremities ultrasound (US) additionally confirmed the presence of a new DVT involving the right POP vein and right posterior tibial vein (PTV) (Figure 4). The patient's treatment plan included initiation of a heparin drip and administration of the EkoSonic Endovascular System (EKOS) before discharge. He was prescribed a regimen of 10 mg of apixaban (Eliquis) twice daily for one week, followed by 5 mg twice daily thereafter.

Five months prior his last admission, the patient once again had experienced another episode of shortness of breath, prompting his outpatient pulmonologist to order a nuclear medicine pulmonary perfusion with a ventilation (V/Q) scan. The results revealed multiple segmental/subsegmental mismatches in both lungs, with perfusion defects exceeding ventilation defects. Subsequent CTA imaging showed a moderate bilateral PE, with thrombi detected in the right upper lobe, right middle lobe, bilateral lower lobe segmental and subsegmental pulmonary arteries, along with a 1.2 cm thrombus in the main pulmonary artery. The Echo indicated an ejection fraction of 55%, moderate right ventricular dilation, and the presence of a mass in the pulmonic valve.

Despite remaining compliant with Eliquis (5 mg twice daily) with no missed doses reported by the patient, this event occurred. The patient underwent another EKOS procedure and was discharged on a bridging regimen of enoxaparin (100 mg twice daily) and warfarin, with instructions to follow up with the warfarin clinic for assessment of successful bridging and to discontinue enoxaparin upon reaching a therapeutic international normalized ratio (INR) level.

Additionally, during the last admission, the patient underwent a thorough blood and coagulation analysis, which included tests for antithrombin antibody, Factor V Leiden mutation, Factor II DNA analysis, lupus anticoagulant, protein C activity, and protein S activity (both total and free antigen). The detailed results of these tests are presented in Table 1.

**Table 1:** Lab findings for the patient (from last admission in 2024) PT: Prothrombin time; INR: International normalized ratio, aPTT: Activated partial thromboplastin time; LC: Liquid chromatography; HDL: High-density lipoprotein; LDL: Low-density lipoprotein; IgG: Immunoglobulin G; IgM: Immunoglobulin M; IgA: Immunoglobulin A; Anticoag Rflx: Anticoagulant reflex test, dRVVT: Dilute Russell’s Viper Venom Time; PTT-LA: PTT level due to lupus anticoagulant; AST: Aspartate aminotransferase *: Abnormal tests **: Low normal to high normal value

Laboratory Investigation	Patients’ Lab Value	Reference Range
White Blood Cell	6.2 x 10^3^ /µL	(3.4-10.8) x 10^3^/µL
Red Blood Cell	5.2 x 10^6^/µL	(3.77-5.28)x10^6^/µL
Hemoglobin	15.7 g/dL	11.1-15.9 g/dL
Hematocrit	45.2%	34.0 - 46.6%
Platelet	94 x 10^3^/µL	150-450 x 10^3^/µL
Total Cholesterol	135 mg/dL	111-199 mg/dL
Triglyceride Level	74 mg/dL	34-150 mg/dL
HDL	45 mg/dL	40-59 mg/dL
LDL	78 mg/dL	<100 mg/dL
PT	19.2 Second*	9.4-11.6 Second
INR	2.1(no unit)*	0.9-1.1 second
aPTT	39 Second*	23-30 second
Troponin TNIH	32.6 ng/L	0-76.2 ng/L
NT-ProBNP	2239 pg/mL*	0-125 pg/mL
Fibrinogen Level	428 mg/dL*	200-400 mg/dL
Glucose	113 mg/dL	70-99 mg/dL
Sodium	139 mmol/L	134-144 mmol/L
Potassium	4.1 mmol/L	3.5-5.2 mmol/L
Chloride	107 mmol/L	96-106 mmol/L
CO_2_	23 mmol/L	20-29 mmol/L
Anion Gap	9 (no unit)	8-12 (no unit)
Blood Urea Nitrogen	17 mg/dL	6-24 mg/dL
Creatinine	0.9 mg/dL	0.57-1.00 mg/dL
Calcium	9.0 mg/dL	8.7-10.2 mg/dL
Total Protein	7.0 g/dL	6.0-8.5 g/dL
Albumin	3.6 g/dL	3.9-4.9 g/dL
Albumin/Globulin Ratio	1.1 (no unit)	1.2-2.2 (no unit)
Total Bilirubin	1.1 mg/dL	0.0-1.2 mg/dL
Alkaline Phosphatase	58 IU/L	44-121 IU/L
AST	16 IU/L	0-40 IU/L
Alanine Aminotransferase	26 IU/L	0-32 IU/L
Estimated Creatinine Clearance	57.2 mL/min/1.73 m^2^	>90 mL/min/1.73 m^2^
B2-Glyco IgG LC	<9 GP1 IgG Units	0-20 GP1 IgG Units
B2-Glyco IgM LC	<9 GP1 IgM Units	0-32 GP1 IgM Units
Cardiolipin IgA LC	<9 APL U/mL	0-11 APL U/mL
Cardiolipin IgG LC	<9 GPL U/mL	0-14 GPL U/mL
Cardiolipin IgM LC	<9 MPL U/mL	0-12 GPL U/mL
dRVVT LC	47.7 Seconds	0.0-47 Seconds
dRVVT Mix LC	40.1 Seconds	0.0-40.4 Seconds
PTT-LA LC	37.9 Seconds	0.0-43.5 Seconds
Lupus Anticoag Rflx LC	Negative	Negative
Protein C-Functional LC	96 %	73-180 %**
Protein S-Functional LC	57% *	63-140%**
Factor II, DNA Analysis LC	Negative	Negative
Protein S Total	56%*	60-150%**
Protein C Total	70%	60-150%**
Protein S Free	79%	61-136%**

**Figure 5:**
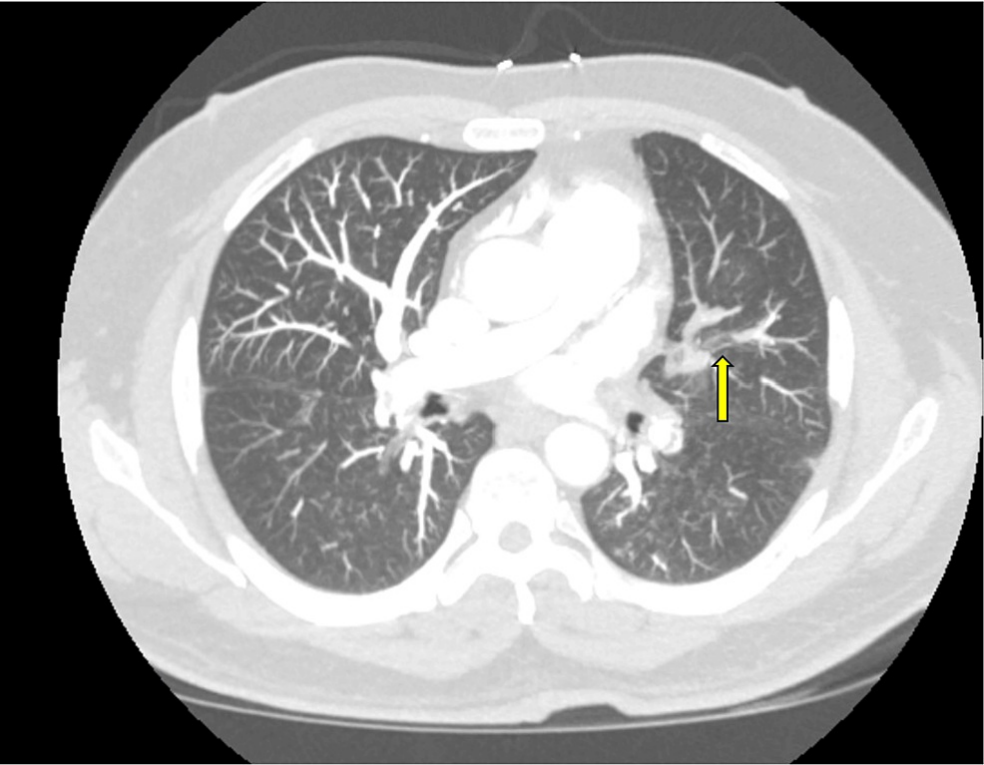
CT angiogram of the chest shows a thrombus in the left main pulmonary artery (arrow) (from last admission in 2024)

He returned to the ED due to few days history of shortness of breath, both with activity and at rest. Upon admission, a CTA revealed a new PE in the left lung, along with residual chronic emboli observed in the central right pulmonary arteries and in the left lower lobe pulmonary arteries (Figure 5)

**Figure 6:**
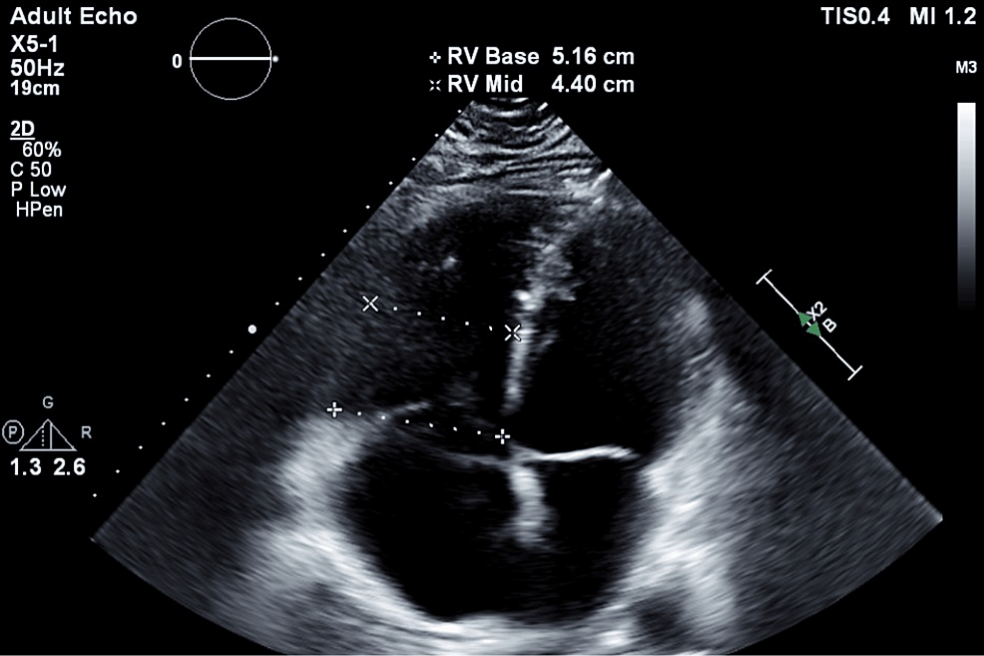
The Echo reveals an enlarged RV and right atrium, along with a flattened interventricular septum (from last admission in 2024) Echo: Echocardiogram; RV: Right ventricle

Additionally, both Echo and CT chest showed mildly enlarged right heart chambers with straightening of the interventricular septum suggesting right heart strain (Figure 6). 

**Figure 7:**
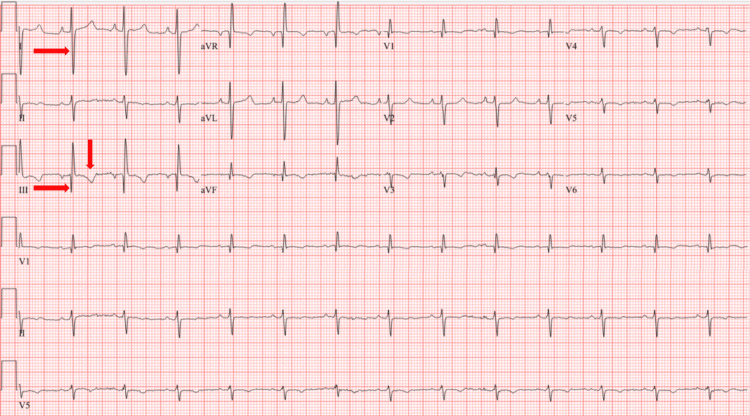
An ECG from the March 2024 admission displayed right axis deviation with a Q1S3T3 pattern (arrows), indicative of right heart strain (observed in approximately 20% of cases of right heart strain) (ECG from his last admission in 2024)

The ECG displayed the classic S1Q3T3 pattern (Figure 7). Echocardiography revealed an ejection fraction of 55% to 60%, along with RV dilation and markedly elevated systolic pressure in the pulmonary arteries. A bilateral US of the lower extremities identified a new thrombosis in the bilateral POP vein and right PTV. The patient reported consistent adherence to warfarin therapy between his discharge in November and the PE recurrence in March 2024, maintaining a therapeutic INR range of 2.2, although his INR upon admission was 2.1.

Initially, the patient was placed on a heparin drip and later transitioned to enoxaparin (90 mg twice daily), yet experienced minimal relief from symptoms of shortness of breath with minimal exertion. Consequently, plans were made to transfer the patient to a tertiary care center for embolectomy. During the most recent admission, a review of past pending labs revealed decreased protein S total levels (58%) and decreased protein S function (60%) from the November 2023 admission. Unfortunately, lab results from other admissions were unavailable, and the patient indicated he had not been previously informed of this deficiency during past admissions or outpatient hematology evaluations.

## Discussion

The clotting mechanism is an evolutionarily developed defense against blood loss in cases of vascular damage. However, flaws in the clotting system are a common occurrence in the medical field. Among these, VTE is the top five most common vascular diseases in the world [3]. VTE consists of DVT and PE. In the United States, the lifetime risk of VTE is estimated to be 8% among adults with a slightly higher rate in men than in women (56 vs 48 per 100,000, respectively) [4,5].

**Figure 8:**
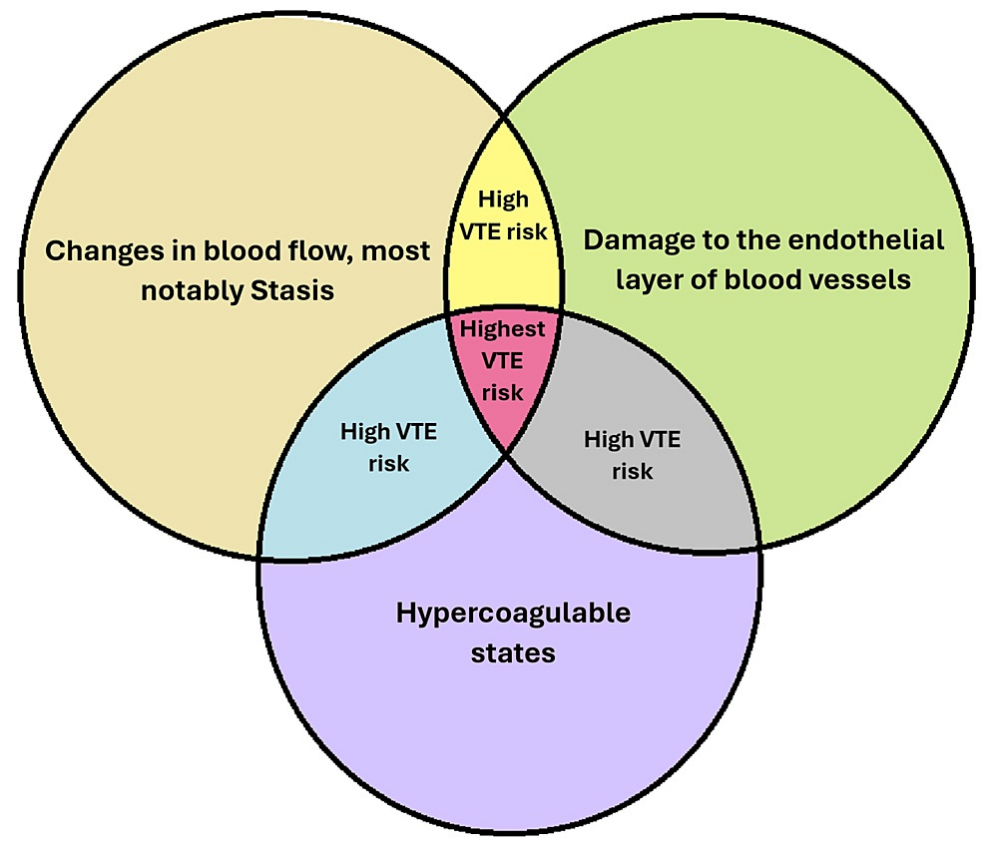
Virchow's triad comprises three factors that predispose a person to develop vascular thrombosis Image created by Ali Ghorbani VTE: Venous thromboembolism; DVT: Deep vein thrombosis

The incidence rises with increasing age, particularly in women, such that PE has an incidence of more than 500 per 100,000 after the age of 75 years. The risk of VTE increases after age 40, and the risk will double for every decade of life afterward. PE is the most dreaded complication of VTE. Since the introduction of D-Dimer (Dimer fragments of fibrin that form during fibrinolysis as a result of intravascular activation of clothing system) and CT scans in the diagnostic approach of DVT and PE, the estimated incidence of PE increased in the general population. However, studies since then have reported stable rates [5,6]. In the United States, PE accounts for approximately 100,000 deaths per year, and in Europe, PE leads to 300,000 deaths annually [7,8]. The primary pathophysiologic cause of VTE is disruption in blood flow, injury to the endothelial layer, or conditions leading to thrombophilia (hypercoagulability). These underlying pathologic changes are termed Virchow’s triad (Figure 8).

The risk factors for VTE, whether DVT or PE, are similar and can be either inherited or acquired. Over 50 genetic risk factors for VTE have been identified, with the most common being factor V Leiden and prothrombin gene mutation [9]. Acquired risk factors can be further classified as provoking or non-provoking. Common provoking risks include recent surgery, trauma, immobilization, initiation of hormone therapy, and active cancer. Common non-provoking factors include obesity and smoking, lack of physical activity and a sedentary lifestyle [9].

Most emboli originate from proximal veins of the lower extremities (iliac, femoral, and POP). Over 50% of patients with proximal vein DVT also present with concurrent PE. DVT of the calf veins seldom embolizes the lungs, and around two-thirds of calf vein thrombi resolve spontaneously after detection [10]. However, if left untreated, one-third of calf vein DVT can extend into the proximal veins, increasing the risk of embolization. PE can also stem from DVT in other veins, such as renal and upper extremity veins, though embolization from these sites is less common [10]. PE remains a leading cause of cardiovascular mortality and morbidity. It most commonly affects the lower lobes and is classified based on severity of clinical presentation as massive, submassive, or low risk (Table 2) [10].

**Table 2:** Classification of PE based on the AHA and ESC [7,8] Table created by Ali Ghorbani. PE: Pulmonary embolism; BP: Blood pressure; RV: Right ventricle; AHA: American Heart Association; ESC: European Society of Cardiology * Troponin and creatin kinase

PE Classification	Major Clinical Findings	Prevalence	30-Day Mortality Rate
Massive (AHA) (high-risk) (ESC)	Presence of hypotension, systolic BP	5%	∼ 65%
Submassive (AHA) (intermediate risk) (ESC)	Normotensive patients with evidence of RV dysfunction or myocardial ischemia on ECG or serum biomarkers*	40%	5–25%
Low Risk	Do not meet criteria of submassive class	55%	<2%

Once a thrombus lodges in the lung, a series of pathophysiological responses follows, including pulmonary infarction, leading to pleuritic chest pain, and abnormalities in gas exchange due to V/Q mismatch, resulting in hypoxemia. This can stimulate respiratory drive, causing hypocapnia and respiratory alkalosis, or if the patient is in shock, it can lead to hypercapnia and respiratory acidosis. Lastly, hypotension resulting from PE is caused by a decrease in stroke volume and cardiac output. In PE patients, pulmonary vascular resistance (PVR) rises due to the physical obstruction of the vascular bed by thrombus and hypoxic vasoconstriction within the pulmonary arterial system. This increased PVR hinders right ventricular outflow, leading to right ventricular dilation and the flattening or bowing of the intraventricular septum. Reduced flow from the RV and RV dilation both decrease left ventricular preload, ultimately compromising cardiac output [10,11].

Clinical presentation

The clinical manifestation of PE correlates directly with its severity. Low-risk PE cases often remain asymptomatic, whereas submassive and massive PE cases tend to exhibit more pronounced symptoms. The spectrum of PE presentation ranges from mild or no symptoms to severe manifestations such as shock, arrhythmia, or sudden death [11,12]. Given that some patients, even those with significant PE, may not display overt symptoms, maintaining a high index of suspicion is vital to avoid overlooking clinically significant cases. Table 3 outlines the most common clinical symptoms observed in patients who do present with symptoms [11,12].

**Table 3:** Most common clinical presentations of PE, which are typically more prevalent in severe cases of PE Table created by Ali Ghorbani PE: Pulmonary embolism; DVT: Deep vein thrombosis, PEA: Pulseless electrical activity

Signs and Symptoms of PE	Frequency in Submassive PE	Frequency in Massive PE
Dyspnea	85%	97%
Chest pain	82%	86%
Tachypnea (>20/min)	81%	87%
Tachycardia	47%	66%
Syncope	85%	81%
Diaphoresis	47%	62%
Signs of DVT	47%	39%
Fever (>37.5˚C)	51%	50%
Hemoptysis	13%	11%
Shock or cardiac arrest (PEA)	<1%	12%

A systematic approach to patients with possible VTE

The initial approach to a patient suspected of having VTE begins with a comprehensive history and thorough physical examination. During the history-taking process, specific attention should be given to potential VTE risk factors, which include recent travel, major surgeries or hospitalizations, the presence of a central venous catheter, myeloproliferative disorders, antiphospholipid syndrome, history of cancer, trauma, smoking habits, pregnancy status, medication use (especially oral contraceptives), previous myocardial infarction or congestive heart failure, spinal trauma leading to lower-extremity paralysis, infections such as Covid-19, and any previous occurrences of VTE. A positive family history of VTE significantly increases the likelihood of VTE in the patient. It is important to assess both acquired and hereditary coagulation disorders in all patients with suspected VTE. Additionally, certain nutritional deficiencies like folate or vitamin B12 deficiency can contribute to thromboembolism.

Furthermore, major inherited thrombophilia including factor V Leiden mutation, prothrombin gene mutation, protein S deficiency, protein C deficiency, and antithrombin deficiency should be evaluated based on the patient's past medical or family history. The lifetime risk of developing VTE is significantly higher in patients with protein S deficiency (such as in this case) (8.5 times higher), antithrombin deficiency (8.1 times higher), protein C deficiency (7.3 times higher), and Factor V Leiden mutation (2.2 times higher) compared to the general population. The overall prevalence of inherited thrombophilia in individuals with DVT ranges from 24% to 37%, which is notably higher compared to about 10% in control groups [13].

During the physical examination, special attention should be given to age, as the risk of VTE increases notably after age 40 and doubles with each subsequent decade thereafter [14]. Additionally, assessing the patient's body mass index is crucial since obesity is a known risk factor for VTE development.

After conducting a thorough history and physical examination, clinicians frequently turn to two main risk stratification tools in clinical practice: the Wells criteria and the pulmonary embolism rule-out criteria (PERC). Although these criteria have overlapping features, PERC is predominantly used in ED settings, while the Wells criteria are more prevalent in ward settings. Table 4 delineates the PERC criteria, while Table 5 provides a breakdown of the Wells criteria [12-15].

**Table 4:** PERC is primarily utilized in the ED. If a patient scores zero, no further workup is necessary as the risk of thromboembolic events is less than 2%, and the patient can be discharged home. PERC: Pulmonary embolism rule-out criteria; PE: Pulmonary embolism, DVT: Deep vein thrombosis; ED: Emergency department

Criteria	No	Yes
Age >50	0	1
Pulse ≥ 100 beats/min	0	1
O_2_ saturation on room air ≤ 95%	0	1
Prior history of DVT/PE	0	1
Recent trauma or surgery	0	1
Hemoptysis	0	1
Exogenous estrogen	0	1
Unilateral leg swelling	0	1
Any of the above present?	No need for further workup as chance is <2%	Further work up is needed

**Table 5:** Modified Wells criteria, widely used in clinical medicine, underwent a change after the addition of the last score (history of documented DVT), resulting in its designation as the modified Wells criteria. Based on it, DVT is likely if the patient score 2 or more, and unlikely if less than 2 Table created by Ali Ghorbani. DVT: Deep vein thrombosis *: This criterion has been added to the original Wells criteria, now recognized as the modified Wells criteria.

Clinical Feature	No	Yes
Active cancer (treatment ongoing or within the previous 6 months or palliative)	0	1
Recently bedridden for more than three days or major surgery within four weeks	0	1
Paralysis, paresis, or recent plaster immobilization of the lower extremities	0	1
Localized tenderness along the distribution of the deep veins	0	1
Entire leg swollen	0	1
Calf swelling by more than 3 cm compared to the asymptomatic leg (measured 10 cm below tibial tuberosity)	0	1
Pitting edema (greater in the symptomatic leg)	0	1
Collateral superficial veins (nonvaricose)	0	1
Previously documented DVT*	0	1

Following an initial assessment and in accordance with the Wells criteria, if further evaluation is warranted, laboratory testing and imaging can be employed to aid in diagnosis and determine the appropriate management for the patient.

Laboratory tests

Routine labs may reveal leukocytosis, elevated erythrocyte sedimentation rate (ESR), increased serum lactate and lactate dehydrogenase (LDH), and elevated levels of aspartate aminotransferase (AST). Additionally, serum creatinine levels are essential to assess the safety of contrast agents used in angiography. Arterial blood gas (ABG) analysis may indicate hypoxemia, although it can be normal in about 18% of patients [14]. Normal ABG readings may also be due to underlying cardiopulmonary conditions in patients with a relevant medical history. Apart from hypoxemia, other common ABG findings include a widened alveolar-arterial gradient for oxygen, respiratory alkalosis with hypocapnia, or acidosis with hypercapnia in patients experiencing shock. While troponin and BNP tests may have limited diagnostic value for PE, they play crucial roles in prognostication and risk stratification for patients with acute PE [15].

An elevated D-Dimer level alone is insufficient to diagnose PE. However, a normal D-Dimer level can effectively rule out PE in individuals with a low likelihood of the condition, obviating the need for further testing. Conversely, an elevated D-Dimer level should prompt additional imaging evaluation. In most patients with an intermediate risk of PE and a normal D-Dimer level, further testing is generally unnecessary. Nevertheless, some experts advocate for imaging in a small subset of these patients due to the higher likelihood of PE, considering the test's low sensitivity. A normal D-Dimer level in high-risk patients is insufficient to exclude PE, rendering this test unnecessary for this group (Table 6) [15].

**Table 6:** The choice of diagnostic tests for patients with PE depends on the severity of clinical presentation and the risk stratification of the patient. In high-risk patients for PE, CTA is the method of choice, and there is no need to check D-Dimer. For low-risk patients, no further testing is necessary, while for those with intermediate risk, D-Dimer can be used to assess the need for CTA. RV: Right ventricle; CTA: CT angiography; Echo: Echocardiogram; SBP: Systolic blood pressure; sPESI: Simplified pulmonary embolism severity index

Patient Risk Level	Clinical findings
Low risk	Normotensive, with no RV dysfunction and no myocardial necrosis/stain
Intermediate-low risk	Normotensive, with RV dysfunction by CT, Echo, or myocardial necrosis/strain
Intermediate-high risk	Normotensive, RV dysfunction by CT or Echo plus myocardial necrosis/strain sPESI >1
High risk	Shock SBP < 90 mmHg for 15 minutes or decreased in SBP 40 mmHg from baseline for 15 minutes

If deemed necessary, CT pulmonary angiography (CTPA) is the preferred imaging method. V/Q scans are suitable for patients unable to undergo CTPA or when it's contraindicated. They may also be used if CTPA results are inconclusive. Lower extremity duplex ultrasound (DUS) can be employed as an initial test for suspected PE. Positive results may warrant initiating anticoagulation therapy, though they do not definitively rule out PE. If Doppler US yields negative results and the patient is at low to intermediate risk for PE, it's generally safe to abstain from anticoagulation and monitor for DVT using serial US until chest imaging is performed. However, if DUS is negative but clinical suspicion remains high, empirical anticoagulation should be considered [16].

CT venography of the lower extremities and pelvis with contrast is seldom used to assess DVT due to its high radiation exposure and should be reserved for specific populations. Magnetic resonance pulmonary angiography (MRPA) is not recommended as the initial test for diagnosing PE but may serve as an alternative imaging option in patients who cannot undergo CTPA or V/Q scans. Concerning the cumulative radiation dose in young or pregnant patients, and with the availability of necessary technology and expertise, MRPA may replace CTPA. However, it is less sensitive and relies more on the technologist's experience [16]. Catheter-based pulmonary angiography, while slightly less sensitive than CTPA, is more invasive and typically reserved for patients requiring concurrent therapeutic interventions. Echocardiograms can aid in diagnosing a fraction of PE cases. In rare instances, a visible thrombus in the proximal pulmonary arteries or a clot in the right heart can assist in PE diagnosis. The emergence of new right heart strain in hemodynamically unstable patients with a high suspicion of PE justifies emergency antithrombotic use in their management. However, in most cases, especially those who are hemodynamically stable, echocardiograms are generally insensitive and nonspecific. Right heart strain can be observed in the echocardiograms of 30-40% of patients with PE, with findings such as increased RV size, decreased RV function, tricuspid regurgitation, abnormal septal wall motion, McConnell's sign, and decreased tricuspid annular plane systolic excursion (TAPSE). An ECG can also be used in the assessment of RV strain. The classic ECG findings of RV strain in ECG is S1Q3T3 pattern which is positive in less than 20% of patients with PE. Dual-energy CT, single-photon emission CT (SPECT), and multi-organ US are being investigated as safer alternatives to CTPA, but not widely available [16].

Differential diagnosis

When patients exhibit signs and symptoms suggestive of PE, key differential diagnoses to consider include heart failure, pneumonia, myocardial ischemia or infarction, pericarditis, acute exacerbations of chronic lung disease, pneumothorax, and musculoskeletal pain. CTPA can help identify several of these alternative diagnoses [16].

Approach to patients with recurrent PE

In recurrent PE cases, a negative D-Dimer result is less common. However, it remains useful in a small percentage of patients (<15%) for differentiating those needing further imaging. It's recommended to have previous imaging available for comparison, as many patients with symptoms resembling their prior PE may not have a new thrombus, highlighting the importance of identifying symptoms caused by a new thrombus [16]. When reviewing past images, consider that thrombi can migrate over time, and clot resolution rates vary. In a study of 79 patients receiving anticoagulant therapy for acute PE, complete clot resolution occurred in 40% within one week, 50% within two weeks, 73% within four weeks, and 81% by four weeks or more. Larger pulmonary arteries resolved clots faster than smaller vessels, especially within the first week [16].

Treatment

PE and DVT represent critical aspects of VTE, ranking as the third most common life-threatening cardiovascular disease in the United States. Anticoagulation stands as the cornerstone of VTE management. In patients with a diagnosis of VTE, the highest risk of recurrent thrombosis and embolization occurs within the initial days and weeks following diagnosis. Therefore, initiating anticoagulation within the first few days (i.e., 0 to 10 days) is crucial in preventing recurrence and VTE-related deaths [16,17].

Many patients with DVT or low-risk PE can receive outpatient treatment using low-molecular-weight heparin alongside a VKA (such as warfarin) or direct-acting oral anticoagulants. Inpatient management of VTE typically commences with parenteral agents, preferably low-molecular-weight heparin. Unfractionated heparin may be necessary in cases of hemodynamic instability, severe renal insufficiency, high bleeding risk, hemodynamic instability, or morbid obesity. Direct-acting oral anticoagulants present an alternative option, albeit with considerations regarding cost and the availability of reversing agents (currently limited to dabigatran, with others under development) [17].

When using warfarin, administration of low-molecular-weight or unfractionated heparin is necessary for at least five days. In the case of warfarin, this concurrent therapy continues until the INR remains at therapeutic level for at least 24 hours. Hemodynamically unstable patients with low bleeding risk may benefit from thrombolytic therapy. However, inferior vena cava filters are not recommended for anticoagulated patients. Standard guidelines recommend a minimum of three months of anticoagulation. However, specific situations such as active cancer or pregnancy may require extended use of low-molecular-weight or unfractionated heparin. Decisions regarding anticoagulation beyond three months should be based on individual risk/benefit assessments.

For symptomatic distal DVT, anticoagulation is warranted, while asymptomatic cases may undergo serial imaging for two weeks before initiating treatment if there is no sign of extension [17].

## Conclusions

As VTEs, especially PEs, continue to be a leading cause of cardiovascular mortality and morbidity, the accurate identification of a PE and prompt initiation of appropriate treatment are paramount. By carefully assessing the patient's clinical presentation and utilizing a discerning approach, along with proper laboratory and imaging studies, clinicians can swiftly and accurately diagnose VTE, assess its severity, and most importantly, determine its source for effective management. Finding the optimal solution can be challenging, especially in patients with acquired or inherited thrombophilia. While studies on the evaluation and treatment of VTE are abundant, there remains a lack of clarity regarding management strategies for patients resistant to medical interventions. This scarcity of evidence is even more pronounced in cases of recurrent VTE and resistance to multiple medical therapies. As demonstrated in this case, there is a growing shortage of evidence-based medical guidance for addressing some patients with recurrent VTE. Are we overlooking a crucial aspect of the coagulation cascade, or do we lack a complete understanding of managing complex scenarios? Are we overlooking a broader perspective? Do we require further studies to establish guideline-directed medical management for individuals experiencing medication failure? These are the questions that remain unclear in medical literature.
